# Tensile Strength of Porcine Aorta Decellularized with Liquefied Dimethyl Ether and DNase

**DOI:** 10.1021/acsomega.2c04103

**Published:** 2022-09-13

**Authors:** Hideki Kanda, Kento Oya, Toshihira Irisawa, Wahyudiono, Motonobu Goto

**Affiliations:** † Department of Materials Process Engineering, Nagoya University, Nagoya, Aichi 464−8603 Japan; ‡ Department of Chemical Systems Engineering, Nagoya University, Nagoya, Aichi 464−8603 Japan; § New Industry Creation Hatchery Center, Tohoku University, 6-6-10 Aoba, Aramaki, Aoba-ku, Sendai, Miyagi 980-8579, Japan; ∥ Super Critical Technology Centre Co. Ltd., Kuwana, Mie 511-0838, Japan

## Abstract

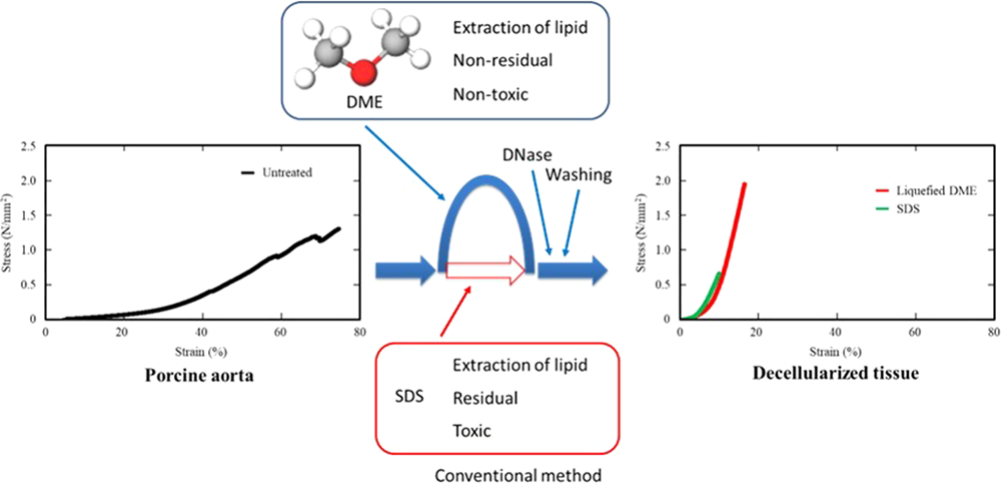

In a previous report, we proposed a method for decellularizing porcine aortas by removing lipids from the aortas using liquefied dimethyl ether (DME) instead of the conventional sodium dodecyl sulfate (SDS). This is followed by DNA fragmentation with DNase. In the current work, the physical properties of porcine aortas decellularized using the DME method are evaluated by tensile strength tests. Conventional SDS decellularized aortas are typically swollen, rupture very easily, and have poor elasticity. By contrast, DME-treated samples are found to be less elastic. However, the maximum stress required for rupture is greater than that for the original aorta. These results indicate that decellularization with DME and DNase increases the maximum stress that can be withstood. Reduction of elasticity may derive from the appearance of temporary C=N bonds due to Schiff-base reactions that occur during the lipid removal process by liquefied DME, and methods to avoid this are desirable.

## Introduction

Aortic aneurysms, aortic stenosis, and atherosclerosis are the leading causes of death worldwide, and aortic transplantation has therefore become a major field. However, because the aorta is critical to survival, a chronic shortage of donors exists. As a result, artificial blood vessels are often employed, but those currently in use have considerable limitations. Therefore, decellularized tissues have been widely studied as a new biomaterial for various tissues and organs.^1−12^ Decellularized tissues are widely accepted because their biocompatibility and regenerative properties are better than those of other non-natural engineered tissues. For decellularized tissues to play a major role in regenerative medicine, the basic properties of decellularized tissues must be investigated. Specifically, understanding their mechanical properties are critical to enable decellularized tissues to replace blood vessels. In this study, the mechanical properties of decellularized porcine aortas prepared by conventional and new methods are compared. The conventional method uses sodium dodecyl sulfate (SDS) with DNase treatment, whereas the new method employs liquefied dimethyl ether (DME) and DNase treatment.

The simplest and most commonly used decellularization method combines lipid removal by SDS and DNA fragmentation by DNase.^1−12^ SDS is a powerful detergent that can effectively remove lipids from tissue. Therefore, when DNase treatment is applied following SDS treatment, DNA can be fragmented efficiently. However, similar to the experience of skin irritation from washing dishes, SDS is a substance that causes inflammation when it remains in decellularized tissues and therefore must be thoroughly removed from decellularized tissues. In addition, fibronectins, glycosaminoglycans, proteoglycans, and extracellular matrix regulators and secreted factors are easily lost during SDS decellularization.^11^

To address these problems in using SDS to remove lipids, in a previous report,^13^ we proposed a method for removing lipids from tissues using liquefied DME as a solvent instead of SDS. We reported that porcine aortas could be decellularized by combining the subsequent DNA degradation process with DNase. In this method, after 1 h of lipid removal using liquefied DME and 3 d of DNA degradation using DNase, the following three criteria for successful decellularization were met: (1) no cell nuclei were observed by microscopic examination of the hematoxylin-eosin-stained decellularized tissue; (2) residual DNA in the decellularized tissue was less than 50 ng/mg-dry; and (3) residual DNA fragments in the decellularized tissue were less than 200 bp.^14,15^

The DME treatment uses highly volatile DME to disrupt the cell membrane and wash out cellular lipids. The liquefied DME treatment involves three steps, as described in the Experimental Section. Briefly, lipids are first extracted from the porcine aorta using liquefied DME under pressure. The lipid-removed porcine aorta is then fragmented with DNA using a DNase solution. Finally, the porcine aorta is washed with water and ethanol to remove DNA fragments. This procedure is the same as the conventional SDS method except that liquefied DME is used instead of SDS. DME is a nontoxic^13^ weakly polar ether with a boiling point of −24.8 °C.^16^ Because of its very low boiling point, DME does not remain in the lipid-removed tissue.^13^ In addition, because it is water-soluble,^17,18^ it can easily extract lipids from wet tissues without the need for drying pretreatment. However, DME is partly mixed with water,^17,18^ and thus when lipids are extracted from the tissue, water is also extracted.^13^

The previous study showed that collagen fibers in the porcine aorta following liquefied DME treatment cross-link because of the dehydration reaction caused by the removal of water. The cross-linked structure produced by the Schiff-base reaction disappears upon treatment with DNase solution, and the Fourier transform infrared spectrum is practically the same as that of the original porcine aorta before liquefied DME treatment.^13^

Whether this history of temporary cross-linking structures results in mechanical changes to the decellularized porcine aorta is unknown. Therefore, in this study, we measured the tensile strengths of two decellularized porcine aortas, one prepared with conventional SDS and DNase treatment and another prepared with liquefied DME and DNase treatment, to determine the ease of fracture and deformation of the decellularized tissues.

## Results and Discussion

**Figure 1:**
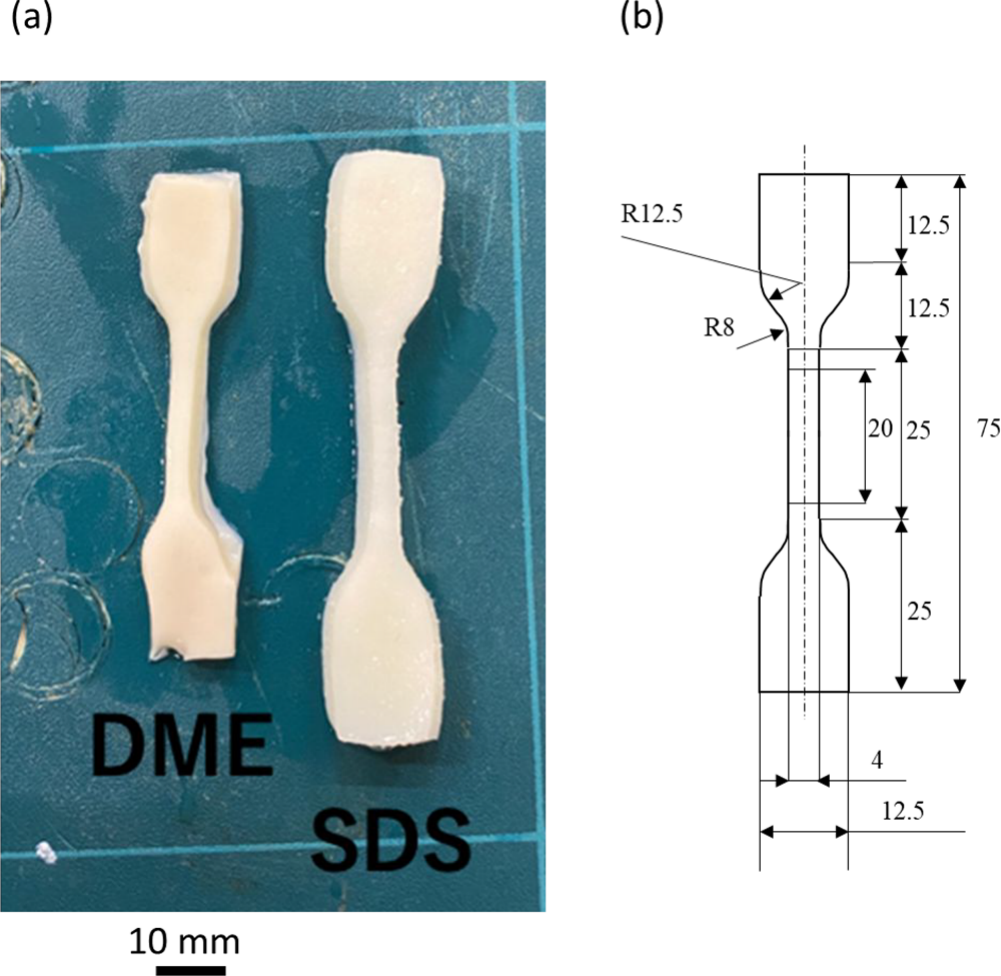
(a) Appearance of porcine aortas following decellularization. (b) Dimensions of the dumbbell-shaped die-cut (reproduced by the authors from the sales company literature).

Figure 1 shows the appearance of decellularized tissue derived from DME and SDS. For comparison, the size of the original porcine aortic tissue is shown at the same scale. The decellularized tissue derived from SDS swelled by 15% in three directions, whereas that derived from DME shrank by 7% from its original size. This was consistent with findings from previous studies: the decellularized porcine aorta tissue derived from DME was densely packed with protein fibers,^13^ whereas large gaps between protein fibers appeared in tissue derived from SDS.^6^ Here, previous studies have reported that arteries have a three-layer (outer, middle, and inner) structure. The outermost layer (also known as the adventia) is surrounded by loose connective tissue consisting mainly of thick bundles of helically arranged collagen fibers. The middle layer (or media) consists of smooth muscle cells, a network of elastic and collagen fibers, and elastic lamellae separating the media into circumferentially isotropic fiber-reinforcing units. The inner layer (or intima) consists mainly of a single layer of endothelial cells, thin basement membrane, and subendothelial layer of collagen fibers.^19,20^ Studies have shown that the fibers are closer to the axial direction in the adventitia, circumferential direction in the media, and somewhere in between these two directions in the intima.^21^ In other words, overall, no significant anisotropy is observed in the fibers that comprise the vessels, and the isotropic expansion in the three directions is consistent with the structure.

**Figure 2:**
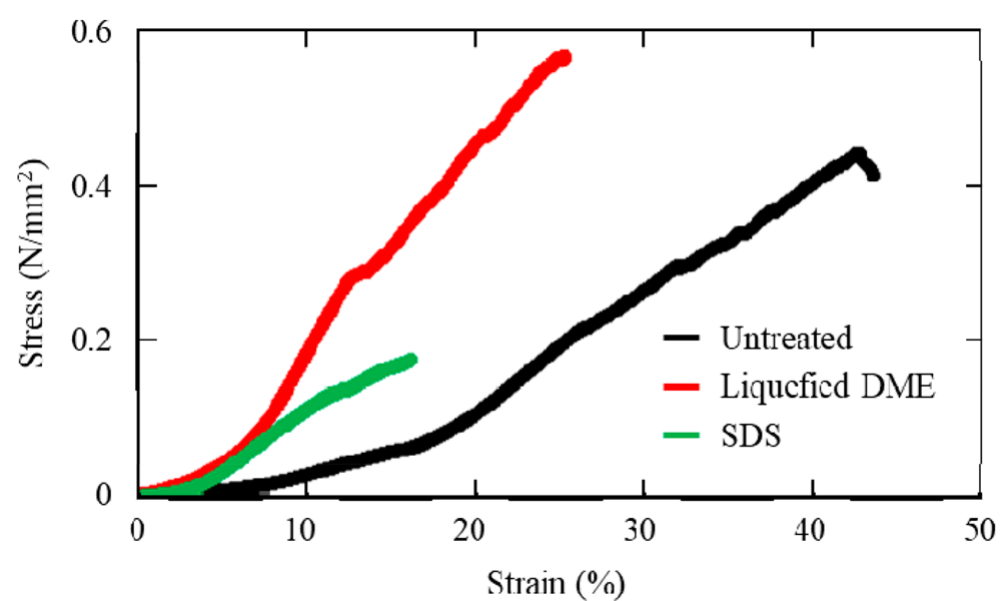
Average strain–stress curves of porcine aortas in the longitudinal direction. Untreated refers to the aorta prior to decellularization treatment. DME: dimethyl ether; SDS: sodium dodecyl sulfate.

**Figure 3:**
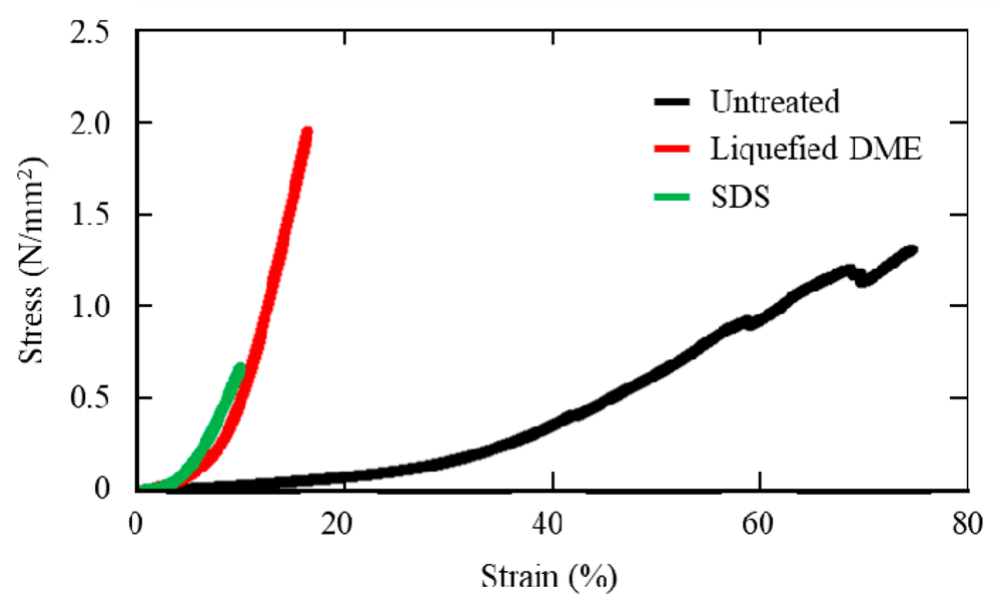
Average strain–stress curves of porcine aortas in the circumferential direction. Untreated refers to the aorta prior to decellularization treatment. DME: dimethyl ether; SDS: sodium dodecyl sulfate.

Results of the tensile test are shown in Figures 2 and 3. Figure 2 shows the results for the sample die-cut along the longitudinal direction, and Figure 3 shows those for the sample die-cut in the circumferential direction.

Figure 2 shows that, in the longitudinal direction, decellularized tissue treated with SDS or liquefied DME was stiffer than the original porcine aorta under the same stress. With the DME treatment, the maximum stress required for rupture was increased as compared to the original porcine aorta, and the strength of the less flexible material was not reduced. However, when SDS was used, it became less flexible as with the liquefied DME and easily ruptured at very weak stresses. This was consistent with an earlier finding that showed that decellularization by SDS altered the tissue microstructure and compromised the biomechanical integrity of the extracellular matrix in exchange for a strong debridement effect.^22^ In addition, the SDS expanded 15% in each direction, as shown in Figure 1. This resulted in a 1/(1.15^2^) = 0.76-fold decrease in the number of fibers per unit area of the stress surface. Figure 2 shows that the maximum stress in the decellularized porcine aorta with SDS was approximately 40% of the original; that is, the strength per fiber decreased to approximately 52.6% (= 0.4/0.76).

The circumferential strain–stress curves of the porcine aorta are shown in Figure 3. The porcine aortas treated with liquefied DME and SDS became less flexible than the original. The maximum stress to rupture was greater when treated with liquefied DME than in the original porcine aorta. When treated with SDS, the maximum stress to rupture was approximately half that of the original porcine aorta. These trends were approximately similar to those in the longitudinal direction.

**Figure 4:**
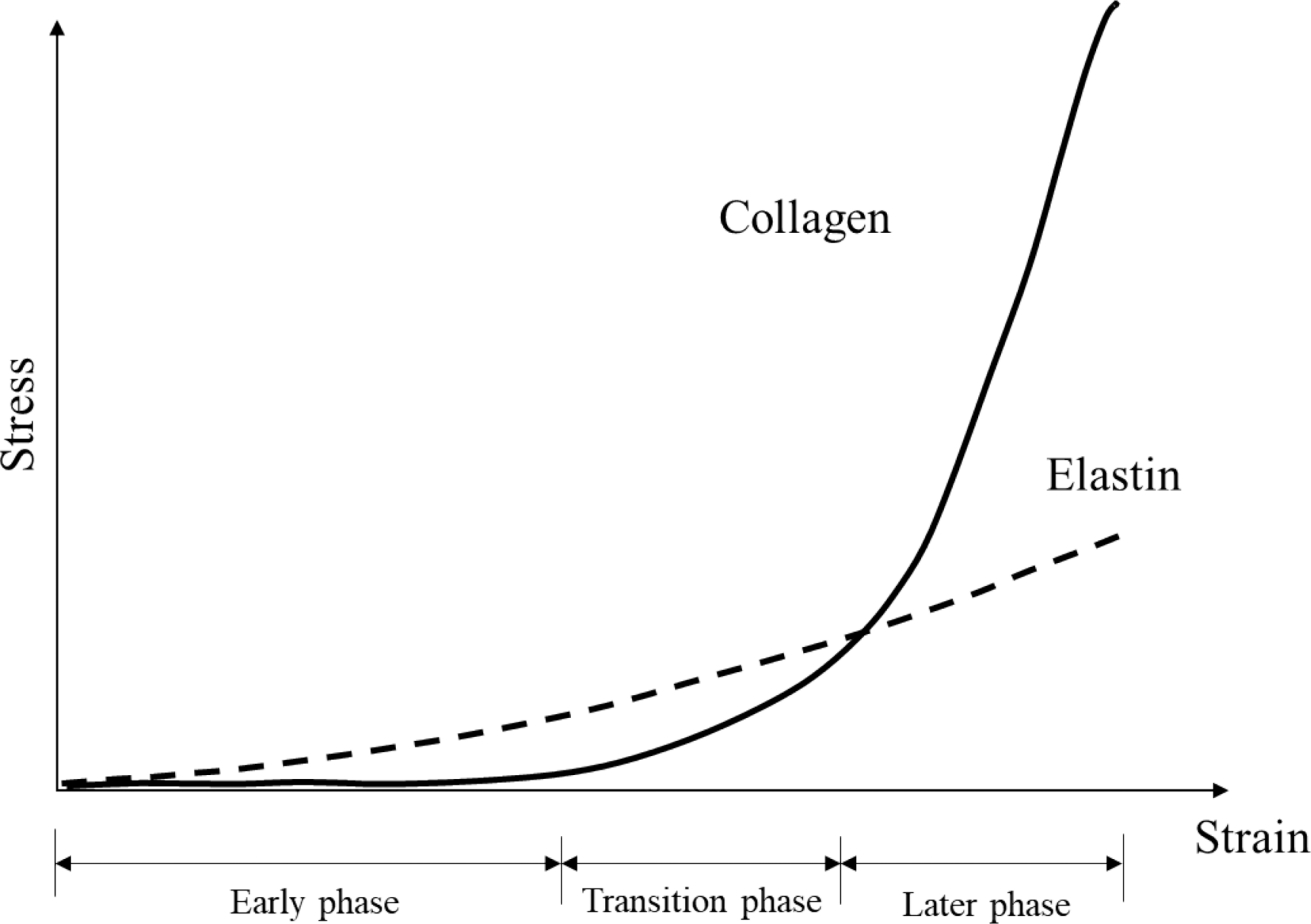
Stress response properties of collagen and elastin when elongated.^23^

In a previous study, vascular tensile testing involved three stages according to the stress strength. In the early phase, when the stress was small, stress was applied to the elastin, whereas stress was hardly applied to the collagen. In the later phase, when the stress was large, stress was mainly applied to the collagen and less to the elastin. The transitional stage was between the early and late stages, when the stress on the elastin was greater than on the collagen. However, most of the incremental stronger stress was on the collagen.^23^ This is illustrated in Figure 4.

On the basis of the findings of previous studies (such as those presented in Figure 4) and when Figures 2 and 3 are considered, the following are possible. First, because of decellularization by liquefied DME, collagen loses its elasticity, which means that the slightest elongation results in high stress. This loss of elasticity may be due to the temporary appearance of C=N bonds caused by the Schiff-base reaction during DME treatment, which may shorten the distance between the collagen and neighboring collagen fibers. This may also change the collagen fibers to a position in which the intermolecular forces with the neighboring collagen fibers are stronger, even at sites in the collagen fibers where no C=N bonds have occurred. In addition, this positional change may remain after the C=N bonds are dissolved by DNase solution treatment. This reduces the movement of the collagen fibers when pulled, resulting in a loss of elasticity. The increased intermolecular forces between collagen fibers also increase the maximum stress just prior to rupture. However, the loss of elasticity is a phenomenon similar to arteriosclerosis, which causes an increase in blood pressure. If animal-derived blood vessels are used such that the Schiff-base reaction does not occur following liquefied DME treatment, the problem of reduced flexibility would be solved.

## Conclusions

In this study, tensile strength tests were conducted on decellularized tissue obtained from porcine aortas by lipid removal with liquefied DME and DNA fragmentation with DNase. Results showed that the tissue with SDS showed poor elasticity and broke under less than half the stress of the original porcine aorta. By contrast, in the case of liquefied DME, the aorta withstood more stress than the original. However, as with SDS, the elasticity was poor and arteriosclerosis was the result. The poor elasticity of artery is most likely due to the temporary formation of C=N bonds by dehydration through the Schiff base reaction during the removal of lipids with liquefied DME.

## Experimental Section

### Materials

Porcine aorta samples for this study were taken from pigs slaughtered for meat processing (and thus were not slaughtered specifically for this experiment) and were obtained from a meat processor (Tokyo Shibaura Organ Co. Ltd., Tokyo, Japan). The porcine aortas were cut into 7.5 cm lengths and used for decellularization studies. The fat on the porcine aortas was trimmed with a knife and stored at 4 °C in phosphate-buffered saline.

To set the samples for tensile strength measurements, both ends of the samples had to be clamped in the measurement device. To prevent stress concentration at the points where they were clamped, the samples were cut using a die-cutting machine (Super Dumbbell SDMP-1000, JIS-K6251-7, Dumbbell Co. Ltd., Saitama, Japan) to the shape shown in Figure 1. The aortas were cut longitudinally or circumferentially before being shaped with the die-cutting machine.

### Decellularized by SDS or DME

The decellularization protocol involves a sequence of three steps: (1) removal of lipids from the porcine aorta by liquefied DME or SDS; (2) fragmentation of DNA by DNase treatment; and (3) washing to remove SDS and fragmented DNA. The only difference between the liquefied DME and SDS treatments is the first step.

#### Liquefied DME Treatment

Liquefied DME treatment was performed according to the protocol derived from a previous study on decellularization.^13^ In brief, a cylindrical column was filled with the porcine aorta, and liquefied DME (Spray Work Air Can 420D, Tamiya, Shizuoka, Japan) was poured into the column at a flow rate of 10 (±1) mL/min for 1 h. Following DME treatment, the inside of the column was depressurized to evaporate the internal DME, and the aorta was removed. The aorta was then shaken in 0.2% DNase solution (Roche Diagnostic, Tokyo, Japan) at 4 °C for 7 d. This was much longer than the 3 d condition required to meet the criteria in the previous study and was done to ensure successful decellularization. Following DNase treatment, the porcine aorta was washed with 80/20 (v/v) ethanol/saline for 3 d.

#### SDS Treatment

SDS decellularization was performed as described in a previous study on decellularization.^2^ In brief, the porcine aorta was washed with 10 mM tris(hydroxymethyl)-aminomethane buffer (Nippon Gene Co., Ltd., Tokyo, Japan) with 0.1% ethylenediaminetetraacetic acid (Nippon Gene Co., Ltd.) and 0.1% SDS for 24 h at room temperature and then thrice rinsed with phosphate-buffered saline. The decellularized tissues were then obtained by treatment with DNase solution and washing as in the DME treatment.

#### Properties of Decellularized Tissue

**Figure 5:**
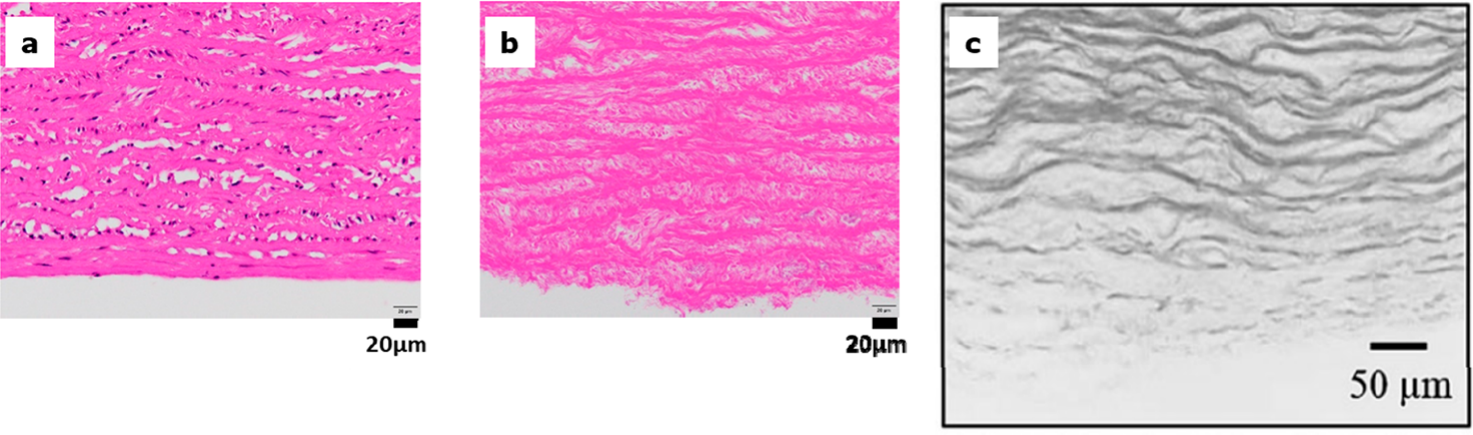
Haematoxylin–eosin staining: (a) Native.^13^ (b) DME extraction and DNase treatment for 7 days.^13^ (c) SDS treatment.^23^

The properties of the decellularized tissue of porcine aorta used in this study are as follows. First, as shown in Figure 5b,^13^ there are no visible cell nuclei by hematoxylin-eosin staining in decellularized porcine aorta treated with DME and DNase for 7 days. This is in stark contrast to the cell nuclei visible in the original porcine aorta shown in Figure 5a.^13^ Also, the DME-treated tissue was dense with protein fibers. On the other hand, previous study has shown that porcine aortas treated with the SDS method have very wide gaps in the protein fibers, as shown in Figure 5c.^23^

**Figure 6:**
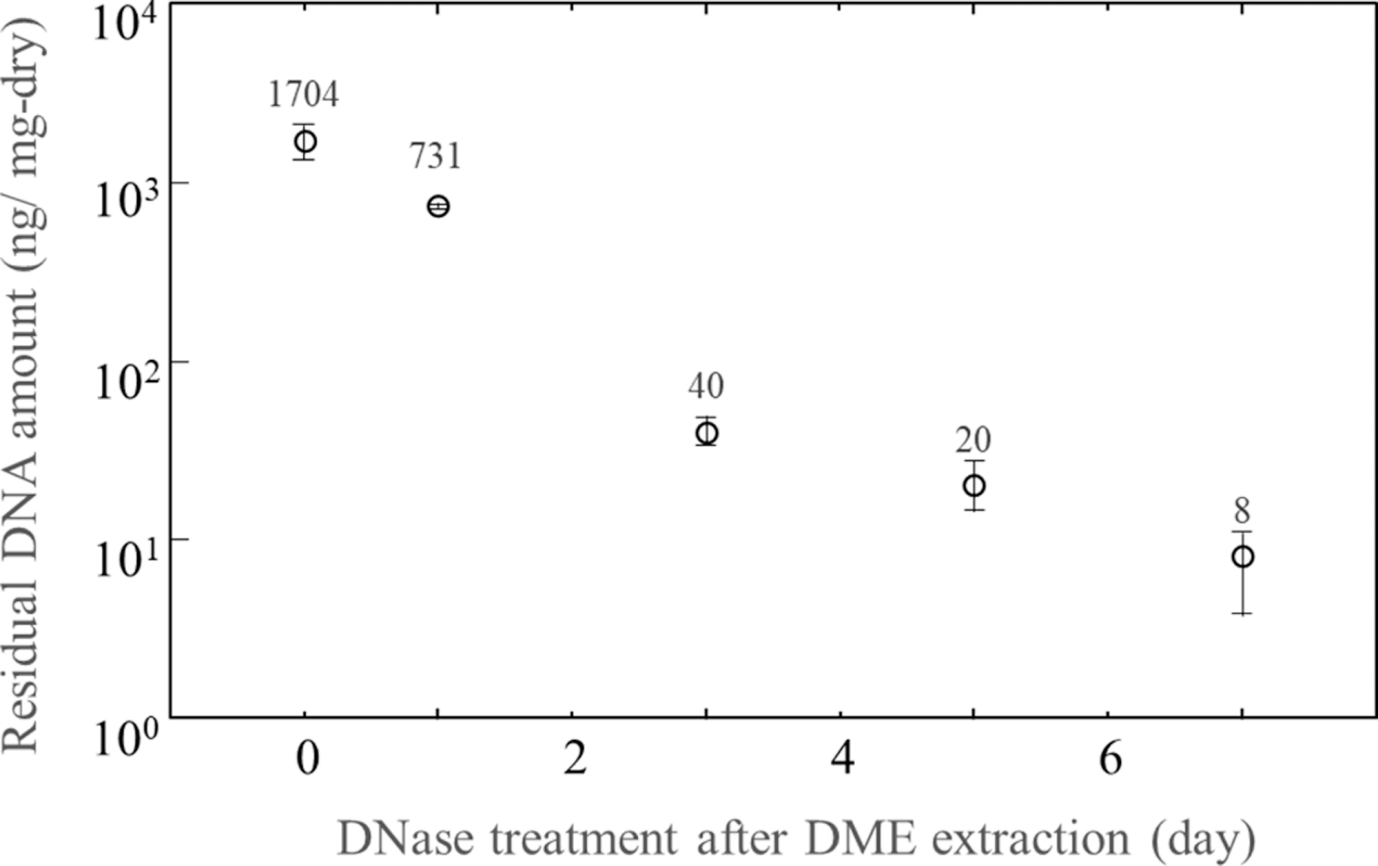
Residual DNA amounts in the porcine aortas.^13^

**Figure 7:**
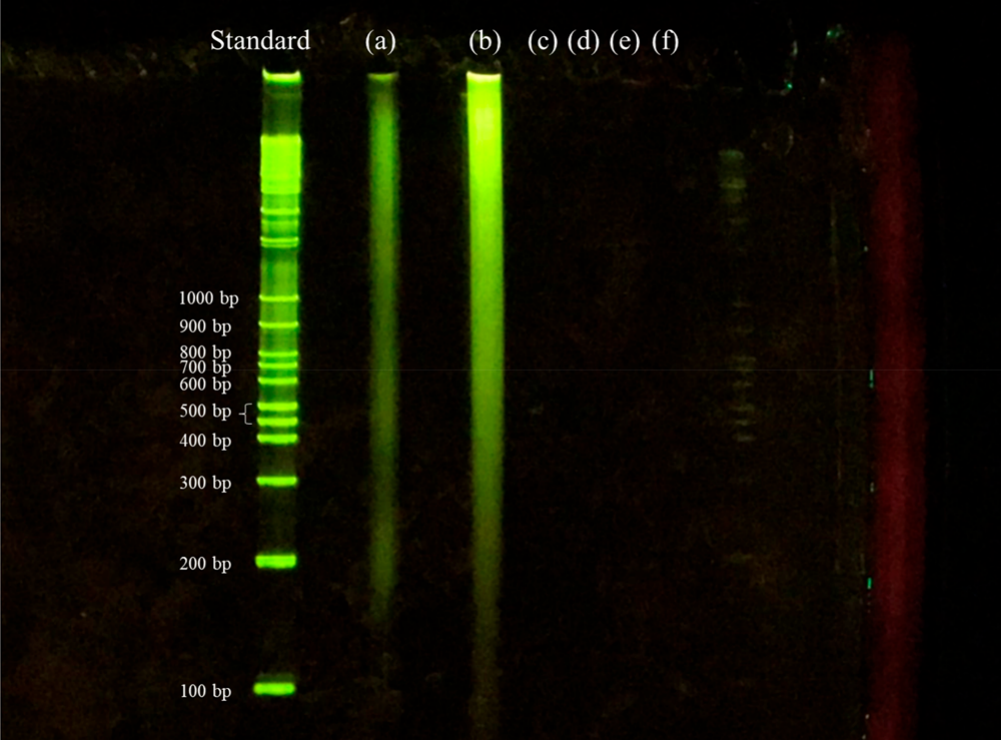
Fragments of residual DNA in the samples detected by agarose gel electrophoresis: (a) Native. (b) DME extraction only. (c-f) DNase treatment for 1 (c), 3 (d), 5 (e), and 7 (f) days following DME extraction.^13^

Also, as shown in Figure 6,^13^ DNA residues were 8 ng/mg-dry in decellularized porcine aortas treated with DME and DNase for 7 days, compared to 1704 ng/mg-dry in the original porcine aorta. In addition, as shown in Figure 7a,^13^ the original porcine aorta was not fragmented at all, while in the decellularized porcine aorta treated with DME and DNase for 7 days, no DNA fragments of 100 bp or more were detected at all as shown in Figure 7f.

Again, the criteria required for decellularized tissue are as follows, as mentioned earlier: “(1) no cell nuclei were observed by microscopic examination of the hematoxylin-eosin-stained decellularized tissue; (2) residual DNA in the decellularized tissue was less than 50 ng/mg-dry; and (3) residual DNA fragments in the decellularized tissue were less than 200 bp”.^14,15^ The decellularized porcine aorta meets these criteria.

### Tensile Strength Measurement

The tensile strengths of the original porcine aortas and decellularized samples were examined based on a previous study.^23^ Stress–strain curves were obtained using a Tensilon universal testing machine (SL-600, Imada-ss Corp., Toyohashi, Japan). Each sample was strained at a rate of 5 mm/min. Seven measurements were performed, and the stress curves obtained from the remaining five tests were averaged, with the results that were both easiest and hardest to fracture being rejected.

## References

[ref1] White J. K.; Agnihotri A. K.; Titus J. S.; Torchiana D. F. A stentless trileaflet valve from a sheet of decellularized porcine small intestinal submucosa. Ann. Thorac. Surg. 2005, 80, 704–707. 10.1016/j.athoracsur.2004.08.063. 16039233

[ref2] Korossis S. A.; Wilcox H. E.; Watterson K. G.; Kearney J. N.; Ingham E.; Fisher J. In-vitro assessment of the functional performance of the decellularized intact porcine aortic root. J. Heart Valve Dis. 2005, 14, 408–421. 15974537

[ref3] Gilbert T. W.; Sellaro T. L.; Badylak S. F. Decellularization of tissues and organs. Biomaterials 2006, 27, 3675–3683. 10.1016/j.biomaterials.2006.02.014. 16519932

[ref4] Prasertsung I.; Kanokpanont S.; Bunaprasert T.; Thanakit V.; Damrongsakkul S. Development of acellular dermis from porcine skin using periodic pressurized technique. J. Biomed. Mater. Res. Part B Appl. Biomater. 2008, 85B, 210–219. 10.1002/jbm.b.30938. 17853423

[ref5] Rosario D. J.; Reilly G. C.; Ali Salah E.; Glover M.; Bullock A. J.; Macneil S. Decellularization and sterilization of porcine urinary bladder matrix for tissue engineering in the lower urinary tract. Regen. Med. 2008, 3, 145–156. 10.2217/17460751.3.2.145. 18307398

[ref6] Funamoto S.; Nam K.; Kimura T.; Murakoshi A.; Hashimoto Y.; Niwaya K.; Kitamura S.; Fujisato T.; Kishida A. The use of high-hydrostatic pressure treatment to decellularize blood vessels. Biomaterials. 2010, 31, 3590–3595. 10.1016/j.biomaterials.2010.01.073. 20149445

[ref7] Flynn L. E. The use of decellularized adipose tissue to provide an inductive microenvironment for the adipogenic differentiation of human adiposederived stem cells. Biomaterials. 2010, 31, 4715–4724. 10.1016/j.biomaterials.2010.02.046. 20304481

[ref8] Kajbafzadeh A. M.; Sabetkish S.; Heidari R.; Ebadi M. Tissue-engineered cholecyst-derived extracellular matrix: a biomaterial for in vivo autologous bladder muscular wall regeneration. Pediatr. Surg. Int. 2014, 30, 371–380. 10.1007/s00383-014-3474-1. 24468716

[ref9] Wolinsky H.; Glagov S. Structural basis for the static mechanical properties of the aortic media. Circ. Res. 1964, 14, 400–413. 10.1161/01.RES.14.5.400. 14156860

[ref10] Hashimoto Y.; Funamoto S.; Sasaki S.; Honda T.; Hattori S.; Nam K.; Kimura T.; Mochizuki M.; Fujisato T.; Kobayashi H.; Kishida A. Preparation and characterization of decellularized cornea using high hydrostatic pressurization for corneal tissue engineering. Biomaterials. 2010, 31, 3941–3948. 10.1016/j.biomaterials.2010.01.122. 20163852

[ref11] Moffat D.; Ye K.; Jin S. Decellularization for the retention of tissue niches. J. Tissue Eng. 2022, 13, 20417314221101151 10.1177/20417314221101151. 35620656 PMC9128068

[ref12] Xing Q.; Yates K.; Tahtinen M.; Shearier E.; Qian Z.; Zhao F. Decellularization of fibroblast cell sheets for natural extracellular matrix scaffold preparation. Tissue Eng. Part C Methods. 2015, 21, 77–87. 10.1089/ten.tec.2013.0666. 24866751 PMC4291209

[ref13] Kanda H.; Ando D.; Hoshino R.; Yamamoto T.; Wahyudiono; Suzuki S.; Shinohara S.; Goto M. Surfactant-free decellularization of porcine aortic tissue by subcritical dimethyl ether. ACS Omega. 2021, 6, 13417–13425. 10.1021/acsomega.1c01549. 34056489 PMC8158793

[ref14] Rana D.; Zreiqat H.; Benkirane-Jessel N.; Ramakrishna S.; Ramalingam M. Development of decellularized scaffolds for stem cell-driven tissue engineering. J. Tissue Eng. Regener. Med. 2017, 11, 942–965. 10.1002/term.2061. 26119160

[ref15] Crapo P. M.; Gilbert T. W.; Badylak S. F. An overview of tissue and whole organ decellularization processes. Biomaterials. 2011, 32, 3233–3243. 10.1016/j.biomaterials.2011.01.057. 21296410 PMC3084613

[ref16] Wu J.; Zhou Y.; Lemmon E. W. An equation of state for the thermodynamic properties of dimethyl ether. J. Phys. Chem. Ref. Data. 2011, 40, 023104 10.1063/1.3582533.

[ref17] Holldorff H.; Knapp H. Binary vapor-liquid-liquid equilibrium of dimethyl ether-water and mutual solubilities of methyl chloride and water: experimental results and data reduction. Fluid Phase Equilib. 1988, 44, 195–209. 10.1016/0378-3812(88)80111-0.

[ref18] Tallon S.; Fenton K. The solubility of water in mixtures of dimethyl ether and carbon dioxide. Fluid Phase Equilib. 2010, 298, 60–66. 10.1016/j.fluid.2010.07.009.

[ref19] Tsamis A.; Krawiec J. T.; Vorp D. A. Elastin and collagen fibre microstructure of the human aorta in ageing and disease: a review. J. R Soc. Interface. 2013, 10, 20121004 10.1098/rsif.2012.1004. 23536538 PMC3645409

[ref20] Gasser T. C.; Ogden R. W.; Holzapfel G. A. Hyperelastic modelling of arterial layers with distributed collagen fibre orientations. J. R. Soc. Interface. 2006, 3, 15–35. 10.1098/rsif.2005.0073. 16849214 PMC1618483

[ref21] Schriefl A. J.; Zeindlinger G.; Pierce D. M.; Regitnig P.; Holzapfel G. A. Determination of the layer-specific distributed collagen fibre orientations in human thoracic and abdominal aortas and common iliac arteries. J. R. Soc. Interface. 2012, 9, 1275–1286. 10.1098/rsif.2011.0727. 22171063 PMC3350738

[ref22] Xu H.; Xu B.; Yang Q.; Li X.; Ma X.; Xia Q.; Zhang Y.; Zhang C.; Wu Y.; Zhang Y. Comparison of decellularization protocols for preparing a decellularized porcine annulus fibrosus scaffold. PLoS One. 2014, 9, e86723 10.1371/journal.pone.0086723. 24475172 PMC3901704

[ref23] Wu P.; Nakamura N.; Kimura T.; Nam K.; Fujisato T.; Funamoto S.; Higami T.; Kishida A. Decellularized porcine aortic intima-media as a potential cardiovascular biomaterial. Interact Cardiovasc Thorac Surg. 2015, 21, 189–194. 10.1093/icvts/ivv113. 25972596

